# Changes in Fecal Carriage of Extended-Spectrum **β**-Lactamase Producing Enterobacterales in Dutch Veal Calves by Clonal Spread of *Klebsiella pneumoniae*

**DOI:** 10.3389/fmicb.2022.866674

**Published:** 2022-06-23

**Authors:** Teresita d.J. Bello Gonzalez, Arie Kant, Quillan Dijkstra, Francesca Marcato, Kees van Reenen, Kees T. Veldman, Michael S. M. Brouwer

**Affiliations:** ^1^Department of Bacteriology, Host-Pathogen Interaction, and Diagnostics Development, Wageningen Bioveterinary Research, Lelystad, Netherlands; ^2^Wageningen Livestock Research, Wageningen University and Research, Wageningen, Netherlands

**Keywords:** veal calves, fecal carriage, *Klebsiella pneumoniae*, clonal spread, extended-spectrum β-lactamase producing Enterobacterales

## Abstract

This study aimed to characterize the changes in fecal carriage of Extended-Spectrum β-Lactamase (ESBL) producing Enterobacterales (ESBL-PE) in a single Dutch veal calves. During the rearing period at the Dutch veal farm, a decrease in fecal carriage of cefotaxime-resistant *Escherichia coli* isolates was observed after 2 weeks at the veal farm, while an increase of cefotaxime-resistant *Klebsiella pneumoniae* isolates was demonstrated. *E. coli* and *K. pneumoniae* were isolated from rectal swabs collected from 110 veal calves in week 2, 6, 10, 18, and 24 after their arrival at the farm. ESBL-PE isolates were selectively cultured and identified by MALDI-TOF. ESBL genes were characterized by RT-PCR, PCRs, and amplicon sequencing. A total of 80 *E. coli* and 174 *K. pneumoniae* strains were isolated from 104 out of 110 veal calves. The prevalence of ESBL-*E. coli* decreased from week 2 (61%) to week 6 (7%), while an unexpected increase in ESBL-*K. pneumoniae* colonization was detected in week 6 (80%). The predominant ESBL genes detected in *E. coli* isolates were *bla*_CTX-M-15_ and the non-ESBL gene *bla*_TEM-1a_, while in *K. pneumoniae bla*_CTX-M-14_ gene was detected in all isolates. Four cefotaxime-resistant *K. pneumoniae* isolates were randomly selected and characterized in deep by transformation, PCR-based replicon typing, and whole-genome sequencing (WGS). The clonal relatedness of a subgroup of nine animals carrying *K. pneumoniae* ESBL genes was investigated by Multi Locus sequence typing (MLST). In four ESBL-*K. pneumoniae* isolates, *bla*_CTX-M-14_ was located on IncFII_K_ and IncFII_NK_ plasmid replicons and the isolates were multi-drug resistant (MDR). MLST demonstrated a clonal spread of ESBL-*K. pneumoniae* ST107. To the best of our knowledge, this is the first study to report a change in fecal carriage of ESBL-PE over time in the same veal calf during the rearing period.

## Introduction

Fecal carriage of antibiotic resistant-bacteria represents an important reservoir for the transmission and dissemination of resistance genes within and between commensal bacteria and to pathogens (Munk et al., 2018). Extended-spectrum β-lactamase (ESBLs) producing Enterobacterales (ESBL-PE) constitutes an important group of multidrug-resistant bacteria reported all over the world in humans and animals (Bush and Fisher, 2011). Over the past years, the potential role of food-producing animals as a reservoir of ESBL genes has been described (European Food Safety Authority (EFSA), 2011; Liebana et al., 2013; Schmid et al., 2013). Moreover, food products contaminated by ESBL-PE have also been identified as a source for the dissemination of antibiotic-resistant bacteria to humans through food consumption and/or manipulation (Overdevest et al., 2011). Likewise, plasmid similarities between ESBL-PE isolates obtained from humans and food-producing animals have been described (Kurittu et al., 2021).

The Enterobacterales family inhabit the gastrointestinal tract of humans and several animal species in a symbiotic relationship. Members of this family, particularly *Escherichia coli* and *Klebsiella pneumoniae*, are commonly associated with a variety of severe infections in humans and animals. In dairy cattle and veal calves, these bacteria can cause mastitis as well as respiratory and gastrointestinal infections (Schukken et al., 2012).

In dairy and veal farms, the use of antimicrobials, particularly third- and fourth-generation cephalosporin β-lactam antibiotics, provides a selective pressure for the emergence of resistant bacteria and the increase of antibiotic resistance by the production of ESBLs (Liebana et al., 2013). The usage of these antimicrobials was greatly reduced in the Netherlands which has led to a reduction in the prevalence of ESBLs (NETMAP_MARAN, 2021). The ESBL genes are commonly located on mobile elements including plasmids, facilitating the dissemination of the antibiotic resistance genes between bacteria (Rozwandowicz et al., 2018). Plasmid-encoded ESBL enzymes inactivate a large variety of β-lactam antibiotics including third-generation cephalosporins such as cefotaxime. The CTX-M family is the most predominant ESBL in Enterobacterales isolates from livestock in Europe (Horton et al., 2011; D’Andrea et al., 2013; Waade et al., 2021). In the Netherlands, the CTX-M-1 group (mainly *bla*_CTX-M-1_ and *bla*_CTX-M-15_) and CTX-M-9 group (mainly *bla*_CTX-M-14_) are the most common ESBLs genes identified in *E. coli* isolates obtained from veal calves (NETMAP_MARAN, 2021).

In a previous study, it was reported that usage of antimicrobials, differences in farm management practices, and the environment contribute to the selection and co-selection of antibiotic resistance in veal calves (Hordijk et al., 2013). In the production system in the Netherlands, veal calves are collected from different dairy farms and mixed before they are distributed among veal farms which provides an ideal scenario for the acquisition and transmission of antibiotic-resistant bacteria among the population of calves. During the rearing period at the veal farm, fecal shedding facilitates the dissemination of antibiotic-resistant bacteria including potential pathogens such as *K. pneumoniae* (Genomic Epidemiology Organization Server, n.d.).

The majority of the studies on the occurrence and prevalence of ESBL-*K. pneumoniae* in dairy cattle and veal calves have been confined within raw milk, food products, and bovine mastitis cases (Dahmen et al., 2013; Diab et al., 2017). Nevertheless, CTX-M genes (*bla*_CTX-M-1,_ *bla*_CTX-M-15,_ and *bla*_CTX-M-14_) detected in diseased calves have also been reported in healthy calves (Pubmlst Server, n.d.; Tshitshi et al., 2020). Despite that, limited data are available on the prevalence of fecal carriage of ESBL-*K. pneumoniae* in veal farms during the rearing period.

Between March 2019 and May 2020, we conducted a large longitudinal study on the prevalence of ESBL-*E. coli* in Netherlands. Rectal swabs were collected from calves born in 13 dairy farms and subsequently transported to 8 veal farms across the country where the animals were followed until slaughter. Samples were collected before transportation of the animals from the dairy farm to the veal farm and subsequently at five different time points at the veal farm (2, 6, 10, 18, and 24 weeks; *manuscript submitted*). In one particular veal farm, a decrease in fecal carriage of cefotaxime-resistant *E. coli* isolates was observed 2 weeks after the arrival of calves at the veal farm, while an increase of cefotaxime-resistant *K. pneumoniae* isolates was demonstrated from week 6 until slaughter.

In the present study, we aimed to: (a) identify the resistance genes in 80 cefotaxime-resistant *E. coli* and 174 cefotaxime-resistant *K. pneumoniae* isolates from veal calves obtained during the rearing period in one particular veal farm; (b) follow-up the fecal carriage of ESBL-*K. pneumoniae* isolates from a subgroup of nine animals and one animal carrying ESBL-*E. coli* over time, to identify the clonal relatedness between the isolates recovered, and (c) identify the mobile elements present in four ESBL-*K. pneumoniae* isolates randomly selected by whole-genome sequencing (WGS)-based analyses.

## Materials and Methods

### *Escherichia coli* and *Klebsiella pneumoniae* Isolates

A total of 80 *E. coli* and 174 cefotaxime-resistant *K. pneumoniae* isolates were identified from 104 out of 110 calves in week 2, 6, 10, 18, and 24 during the rearing period at the veal farm, see Marcato et al. (2022) for the complete experimental setup. The remaining six animals were culture negative during all time points. In brief, rectal swabs were placed in 3 ml of Buffer Peptone Water (BPW; Becton Dickinson GmbH, Heidelberg, Germany) and incubated overnight at 37°C. After incubation, an aliquot (10 ul) of the enriched solution was plated on MacConkey agar plates with 1 mg/L of cefotaxime and incubated overnight at 44°C (European Reference Laboratory-Antimicrobial Resistance, n.d.).^1^ A single random pink colony was streaked onto Heart Infusion Agar (HIS; Becton Dickinson GmbH, Heidelberg, Germany) supplemented with 5% sheep blood and incubated at 37°C for 24 h to obtain a pure culture. Bacterial isolates were subsequently identified by Matrix-Assisted Laser Desorption Ionization-Time of Light mass spectrometry (MALDI-TOF MS; Bruker Daltonik, Germany). All the isolates were preserved at −80°C for further analysis.

### Molecular Identification of *Escherichia coli* and *Klebsiella pneumoniae* ESBL Encoding Genes

The *E. coli* and cefotaxime-resistant *K. pneumoniae* isolates were further analyzed by a Real-time PCR assay on a light cycler System (Applied Biosystems, 7500 Fast Real-Time PCR System) for the detection of ESBL genes *bla*_CTX-M-1_ group, *bla*_CMY_, *bla*_TEM,_ and *bla*_SHV_ as previously described, using bacteria cell boiled lysate method as DNA template (Geurts et al., 2017; Veldman et al., 2018). In case of negative results, single PCRs for *bla*_CTX-M-2_ group, *bla*_CTX-M-8/25_, *bla*_CTX-M-9_ group and chromosomal *bla*AmpC were performed (Dierikx et al., 2012; Liakopoulos et al., 2016a). Well-defined strains with known ESBLs genes were included as positive controls in the PCR assays. The identification of the ESBL detected by PCR was confirmed by DNA Sanger sequencing using the PCR product by QIAquick^®^ PCR Purification kit (Qiagen^®^). Subsequently, the PCR product was purified using Sephadex (Merck) and used for the DNA Sanger sequencing (3130 Genetic Analyzer) as previously described (Liakopoulos et al., 2016a). The sequences were compared with reference sequences obtained from GenBank using the Sequencher 5.4.6 software.

### *Klebsiella pneumoniae* Antimicrobial Susceptibility Testing

Antimicrobial susceptibility tests of four cefotaxime-resistant *K. pneumoniae* isolates randomly selected from the earliest and latest time point possible (*n* = 2 week 6 and *n* = 2 week 24) were tested by broth microdilution using standard European antibiotic panels EUVSEC and EUVSEC2 (Thermo Fisher, “Sensititre^™^ Gram-Negative MIC Plate” n.d.) (World Health Organization (WHO), n.d.). *E. coli* ATCC 25922 was used as a control reference strain. The results were interpreted using the EUCAST ECOFFs (v7.1),^2^ in case epidemiological cut-off values (ECOFFs) for *K. pneumoniae* were lacking, we used *E. coli* ECOFFs for the interpretation. The *K. pneumoniae* isolates, two from week 6 and one from week 24, were obtained from animals that were initially colonized with ESBL-*E. coli* at week 2 carrying *bla*_CTX_M-15_ and non-ESBL *bla*_TEM-1a_ gene, while the additional *K. pneumoniae* isolates included from week 24 were colonized with *K. pneumoniae* in week 6 and 10 and negative culture in week 2.

### *Klebsiella pneumoniae* Plasmid and WGS Analysis

The same four ESBL-*K. pneumoniae* isolates used for antimicrobial susceptibility testing were characterized in depth using molecular methods. Plasmids carrying ESBLs genes were extracted from pure culture using a miniprep method and transformed by electroporation into competent DH10B cells (Thermo Scientific, United States) as previously described (Liakopoulos et al., 2016a). The obtained transformants were selected on Luria Bertani (LB) agar plates supplemented with cefotaxime (1 mg/L) and confirmed for the presence of the ESBL gene using PCR. The plasmid typing was performed using the PCR-Based Replicon Typing (PBRT) 2.0 Kit (DIATHEVA, Fano, Italy) as previously described (Carattoli et al., 2005). To confirm the location of the identified genes on the plasmids in the four *K. pneumoniae* selected isolates, WGS was performed. The *K. pneumoniae* DNA was isolated and purified using the Qiagen Blood and tissue DNA isolation kit, and DNA concentration was measured with a CLARIOstar Plus (BMG Labtech). The isolated DNA was used for library preparation using the KAPA HyperPlus Kit (KAPA BIOSYSTEMS). DNA was loaded onto the MiSeq (Illumina) sequencer using the MiSeq Reagent kit v3 (Illumina) with pair-end reads, generating 250–300-bp read length.

In addition, MinION long read sequencing was performed using a single *K. pneumoniae* isolate as representative of the other three isolates randomly selected. DNA extraction was conducted using the Gentra Puregene Blood Kit (Qiagen). The preparation of the DNA for sequencing using 500 ng of purified DNA was performed using the Genomic DNA Ligation kit (SQK-LSK109, Oxford Nanopore Technologies, United Kingdom). The DNA sample was barcoded using the Native barcoding genomic DNA kits (EXP-NBD104 Oxford Nanopore Technologies, United Kingdom). The run of the samples was performed in a Flongle flow cell (FLO-FLG001, Oxford Nanopore Technologies, United Kingdom; Software v19.06.8). Base-calling was set on High-Accuracy base calling, and adapter trimming was performed through Porechop v0.2.3. After demultiplexing and adapter trimming, the Hybrid assemblies of short sequencing reads of the four randomly selected isolates and long-read sequencing reads of the single selected representative isolate were performed using Unicycler v0.4.7. The assembled genome was analyzed using tools from the “Center for genomic Epidemiology” (CGE) website.^3^

### *Klebsiella pneumoniae* and *Escherichia coli* Multi Locus Sequencing Typing

A subgroup of nine animals (*n* = 27 isolates) carrying ESBL-*K. pneumoniae* were followed-up over time to determine whether or not these ESBL-*K. pneumoniae* isolates were clonally related (Table 1). Multi Locus Sequencing Typing (MLST) was carried out according to the protocol previously described in the pubmlst web server.^4^ The sequence obtained was compared with the sequences available on the Genomic Epidemiology website. In addition, the only animal colonized with ESBL-*E. coli* (*n* = 4 isolates) over time along with the ESBL-*K. pneumoniae* isolates was also included in the MLST analysis. No additional ESBL-*E. coli* isolates were selected for MLST analysis.

**Table 1:** Distribution of fecal carriage of ESBL-*Klebsiella pneumoniae* isolates per time point (week 6 until week 24 after arrival of calves at a Dutch veal farm) selected for MLST analysis.

Animal ID	Week 6	Week 10	Week 18	Week 24
1	*K. pneumoniae*	*K. pneumoniae*		*K. pneumoniae*
2	*K. pneumoniae*	*K. pneumoniae*		*K. pneumoniae*
21	*K. pneumoniae*	*K. pneumoniae*		*K. pneumoniae*
28	*K. pneumoniae*	*K. pneumoniae*	*K. pneumoniae*	
37	*K. pneumoniae*	*K. pneumoniae*		*K. pneumoniae*
39	*K. pneumoniae*		*K. pneumoniae*	*K. pneumoniae*
59	*K. pneumoniae*	*K. pneumoniae*	*K. pneumoniae*	
70	*K. pneumoniae*	*K. pneumoniae*	*K. pneumoniae*	
103	*K. pneumoniae*	*K. pneumoniae*	*K. pneumoniae*	

### Accession Number

The Illumina (NGS) sequence data sets generated and analyzed in this study have been deposited in the European Nucleotide Archive (ENA) at EMBL-EBI under accession number PRJEB50519.

### Statistical Analysis

A *t*-test assuming equal variance statistical test was performed to indicate if a significant change was observed over time between animals colonized with cefotaxime-resistant *E. coli* and *K. pneumoniae.*

## Results

**Figure 1:**
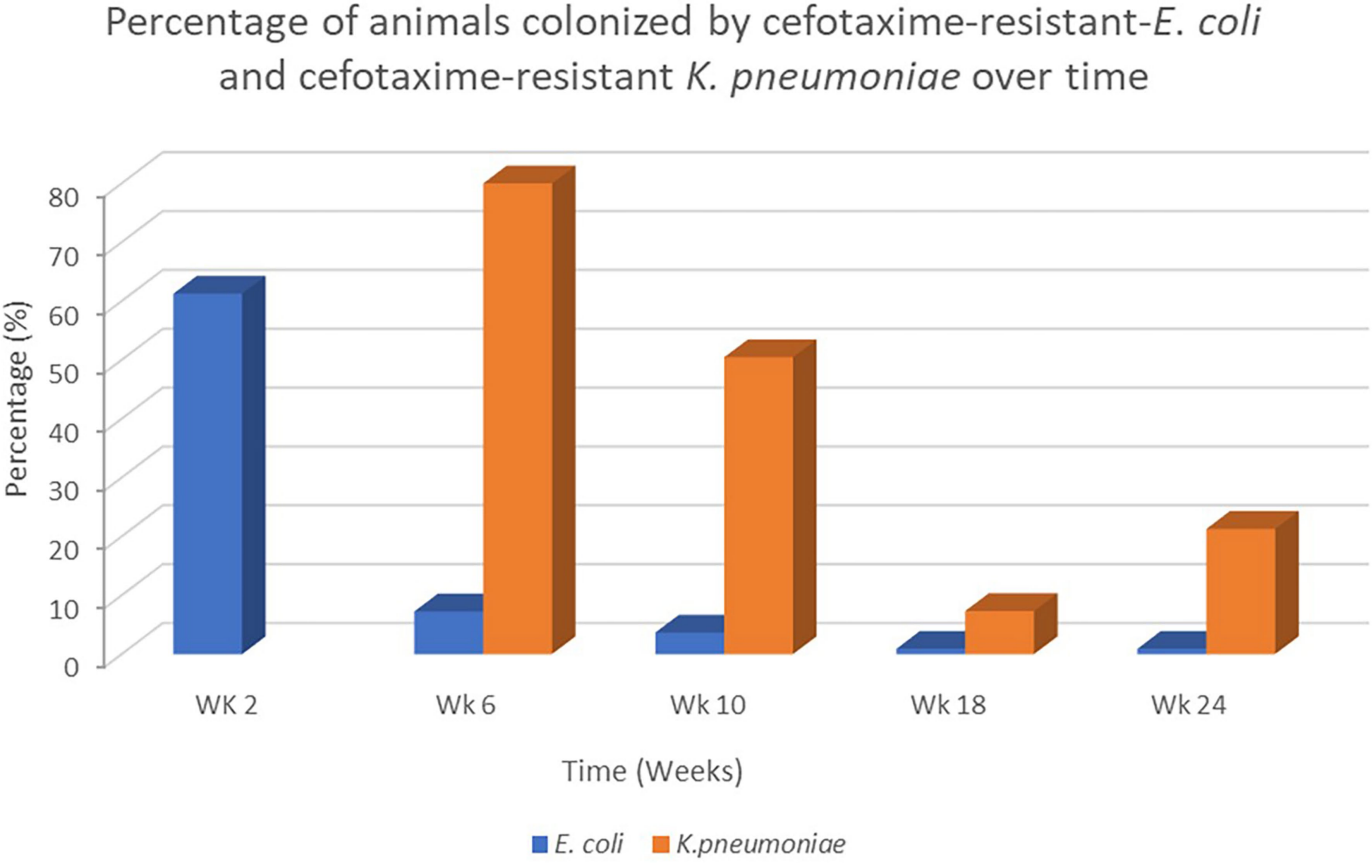
Percentage of animals colonized by cefotaxime-resistant *Escherichia coli* and *Klebsiella pneumoniae* isolates recovered over time during the rearing period at the veal farm from week 2 after arrival of calves at the veal farm until week 24.

Veal calves were followed longitudinally from the dairy farms to the veal farm as previously described. A total of 80 *E. coli* and 174 cefotaxime-resistant *K. pneumoniae* isolates from rectal swabs obtained from 104 out of 110 veal calves located in the same veal farm in the Netherlands were analyzed. In this particular farm, a decrease of fecal carriage of cefotaxime-resistant *E. coli* isolates was observed from week 2 after transportation of calves from the dairy farms to the veal farm (61.3%) to week 6 at the veal farm (7.3%). Instead, an increase of fecal carriage of cefotaxime-resistant *K. pneumoniae* isolates was observed from week 6 until week 24 (Figure 1; *p* > 0.05). We did not detect cefotaxime-resistant *K. pneumoniae* isolates previous to week 6. The prevalence of cefotaxime-resistant *K. pneumoniae* isolates fluctuated during the rearing period, where the highest prevalence was detected in week 6 (80%) and the lowest in week 18 (7.3%). Calves were individually treated with antibiotics [β-Lactam (ampicillin, benzylpenicillin), amphenicol (florfenicol) and, aminoglycoside (gentamicin)] in week 2 (*n* = 18), week 6 (*n* = 32), week 10 (*n* = 78), week 18 (*n* = 31) and week 24 (*n* = 30) due to a respiratory disease (unspecified) for at least 8 weeks. In addition, a nonsteroidal anti-inflammatory drug was given in combination with the antibiotics during the same period. Three batch antibiotic treatments at herd level included tetracycline and aminoglycosides which were provided *via* the milk for 10 feedings within the first 6 weeks after arrival to the veal farms. Since the differences in the production system and management were considered minimal between all the veal farms, no data for risk factor analysis were included at this level.

### Molecular Identification of *Escherichia coli* and *Klebsiella pneumoniae* ESBL Encoding Genes

The *bla*_CTX-M-1_ group and *bla*_TEM_ genes were detected by RT-PCRs in all of the cefotaxime-resistant *E. coli* isolates. The sequencing results showed that *bla*_CTX_M-15_ was present in all the *E. coli* isolates and in combination with the non-ESBL *bla*_TEM-1a_ in 74 *E. coli* isolates (Supplementary Table 1), the other six *E. coli* isolates contains only the *bla*_CTX-M-15_ gene.

Furthermore, the *bla*_SHV_ and *bla*_TEM_ were detected by RT-PCRs in all the cefotaxime-resistant *K. pneumoniae* isolates. The sequencing results showed that a non-ESBL allelic variant identified as *bla*_TEM-1b_ and a novel chromosomal variant sharing 99.5% identity to *bla*_SHV-1-8_ and other variants was identified, which results in a synonymous amino acid sequence. We also obtained positive PCR products for *bla*_CTX-M-9_ and *bla*_CTX-M-14_ genes_._ The sequencing analysis indicates that the fragment sequence corresponds to *bla*_CTX-M-14_. Furthermore, all the cefotaxime-resistant *K. pneumoniae* isolates were tested by PCR and the sequencing results indicates the presence of the *bla*_CTX-M-14_ in all the isolates (Supplementary Table 1).

The antimicrobial susceptibility test of four randomly selected *K. pneumoniae* isolates showed an identical resistance profile. The isolates were susceptible to colistin, carbapenems (imipenem, meropenem, ertapenem), azithromycin, chloramphenicol, nalidixic acid, tigecycline, and resistant to ciprofloxacin, gentamicin, sulfamethoxazole, trimethoprim, and tetracycline (Table 2). The ESBL phenotype was confirmed by showing resistance to cefotaxime and ceftazidime, susceptibility to cefoxitin, and synergy with clavulanic acid in combination with cefotaxime and ceftazidime.

**Table 2:** Antimicrobial susceptibility testing expressing the Minimal Inhibitory Concentration (MIC) determined in four *Klebsiella pneumoniae* isolates collected from calves in week 6 and week 24 at the veal farm.

Isolate ID	63_wk6	68_wk6	2_wk24	37_wk24
EUVSEC^*^ panel		MIC(mg/L)		
Ampicillin	>64	>64	>64	>64
Azithromycin	16	16	16	16
Cefotaxime	>4	>4	>4	>4
Ceftazidime	2	1	2	2
Chloramphenicol	≤8	≤8	≤8	≤8
Ciprofloxacin	1	1	1	0.5
Colistin	≤1	≤1	≤1	≤1
Gentamicin	>32	>32	32	>32
Meropenem	≤0.03	≤0.03	≤0.03	≤0.03
Nalidixic acid	8	8	8	8
Sulfamethoxazole	>1,024	>1,024	>1,024	>1,024
Tetracycline	>64	>64	>64	>64
Tigecycline	1	1	0.5	1
Trimethoprim	>32	>32	>32	>32
EUVSEC2^**^ panel				
Cefepime	4	8	4	8
Cefotaxime	64	32	64	64
Cefotaxime/clavulanic acid	≤0.06/4	≤0.06/4	0.12/4	0.12/4
Cefoxitin	4	4	4	4
Ceftazidime	2	2	2	2
Ceftazidime/clavulanic acid	0.25/4	0.25/4	0.25/4	0.25/4
Ertapenem	≤0.015	0.06	0.03	0.03
Imipenem	0.25	0.25	0.25	0.5
Meropenem	≤0.03	≤0.03	≤0.03	0.06
Temocillin	4	8	4	16

* EUVSEC, EU Surveillance Salmonella/*Escherichia coli*.

** EUVSEC2, EU Surveillance ESBL.

### *Klebsiella pneumoniae* Plasmid and WGS Data Analysis

Transformation experiments were performed using competent *E. coli* to determine if the *bla*_CTX-M-14_ gene was present on a transferable plasmid. Using the four ESBL-*K. pneumoniae* isolates showed a successful transferability of the *bla*_CTX-M-14_ gene. The plasmid-based replicon typing analysis detected the presence of two replicons in the transformants: IncFII_K_ and IncFIB_KN_ type. Hybrid analysis of long-short read sequencing showed that the *bla*_CTX-M-14_ gene and the plasmids replicons previously identified were located in a 258.971 bp contig in *K. pneumoniae* isolates 2 week 24. The other three isolates were sequenced with short-read sequencing only which resulted in *bla*_CTX-M-14_ and the plasmid replicons in separate smaller contigs. Additionally, plasmid-mediated quinolone resistance (PMQR) genes *qnrS1* and *OqxAB* were identified in the four *K. pneumoniae* isolates, along with genes conferring resistance to aminoglycosides, sulfamethoxazole, fosfomycin, tetracycline, trimethoprim and quaternary ammonium compound-resistance protein (Table 3).

**Table 3:** Molecular profile of fecal carriage of ESBL-*Klebsiella pneumoniae* obtained from calves during the rearing period at a Dutch veal farm.

Antibiotic family	*Resistance gene*	% Identity	Accession no.	ST	Replicon type
Beta-lactams	*bla_CTX-M-14_*	100	AF252622	107	IncFIB_k_, IncFIB_kn_
	*bla_TEM-1B_*	100	AY458016		
	*bla_SHV-1-8_*	99.5	GQ407137		
Aminoglycoside	*aph(6)-Id*	100	M28829		
	*aph(3″)-Ib*	100	AF321551		
	*aac(3)-IId*	99.8	EU022314		
Sulfonamides	*Sul1*	100	U12338		
	*Sul2*	100	AY034138		
Fosfomycin	*Fos*A	96.4	ACZD01000244		
Tetracycline	*tet* (D)	100	AF467077		
	*tet* (A)	100	AJ517790		
Trimethoprim	*dfr*A*1*	100	X00926		
Quinolones	*qnr*S*1*	100	AB187515		
Efflux pump	*Oqx*A	99.4	EU370913		
	*Oqx*B	99.4	EU370913		
QACs	*qac*E	100	X68232		

The molecular profile was identical in the four *Klebsiella pneumoniae* isolates sequenced.

### ESBL-*Klebsiella pneumoniae* Clonality Screening by MLST

MLST analysis was carried out on a set of 27 ESBL-*K. pneumoniae* isolates from nine veal calves that were colonized at multiple time-points to determine the clonality of the *K. pneumoniae*. All 27 ESBL-*K. pneumoniae* isolates presented an identical sequence type, all belonging to ST 107. Similarly, all the 4 ESBL-*E. coli* isolates obtained from a single animal colonized at the same rearing period as the animals colonized by ESBL-*K. pneumoniae* present an identical sequence type, all belonging to ST 46.

## Discussion

The World Health Organization (WHO) has included third-generation cephalosporin-resistant *E. coli* and *K. pneumoniae* into the group of “critical pathogens” due to the increasing challenges for infection treatment, highlighting the importance of the monitoring and prevention of infections in humans and animals. In humans, Meijs et al. (2021) have recently reported a high prevalence of fecal carriage of ESBL-*E. coli* and ESBL-*K. pneumoniae* in veterinary healthcare workers in the Netherlands compared to the general Dutch population (9.8% and 5%, respectively) (Meijs et al., 2021). Likewise, in Dutch hospitals, the prevalence of ESBL-*K. pneumoniae* in 2020 increased up to 15% compared to 2019 (12%) (NETMAP_MARAN, 2021). In the longitudinal study reported in the present manuscript, an unexpected increase in fecal carriage of ESBL-*K. pneumoniae* was detected in veal calves. This increase occurred in one single farm, 6 weeks after arrival of the calves to the veal farm and occurred along with a decrease in ESBL-*E. coli* which is typical in many farms at this time point (*submitted manuscript*). A previous study showed a similar low prevalence of ESBL-*E. coli* after 10 weeks at the veal farm (Hordijk et al., 2013). Previously, Adler et al. (2015) showed that the prevalence of ESBL-PE was high in calves (age, < 4 months) compared to adult cows (age > 25 months) (Adler et al., 2015). At the slaughterhouses, a high prevalence of ESBL producers has been reported in France (29.4%) (Haenni et al., 2014) and Switzerland (25%) (Geser et al., 2012), whereas a shift in enteric bacteria species carrying ESBL producers in the same calves has not been reported previously. Therefore, we assessed the characterization of fecal carriage ESBL-*E. coli* and ESBL-*K. pneumoniae* isolates from selected veal calves and the distribution of the ESBL genes present in the calves’ population over time.

In our study, the *bla*_CTX-M-15_ was the dominant ESBL gene detected in *E. coli*. In the Netherlands, the percentage of ESBL-*E. coli* reported in calves at slaughter age was 47% in 2018 and decreased to 38.1% in 2020, in which the CTX-M-1 group was the most dominant ESBL type detected (NETMAP_MARAN, 2021). The global spread of *bla*_CTX-M-15_ has been associated with particular *E. coli* clones such as clonal complex ST131 (Nicolas-Chanoine et al., 2014) and ST46 (Mshana et al., 2011). The *E. coli* clone ST46 harboring *bla*_CTX-M-15_ was detected in our study. The ST46 harboring *bla*_CTX-M-15_ has been previously identified in healthy chickens and pigs in Nigeria (Chah et al., 2018), in bovine feces in Tunisia (Hassen et al., 2019) and in the aquatic environment in Bangladesh (Rashid et al., 2015), suggesting that ESBL_*E. coli* clonal spread occurs in different environments across the world.

Furthermore, in the *K. pneumoniae*-cefotaxime resistance isolates in our study, the *bla*_CTX-M-14_ gene was identified in all the isolates. From those, four isolates were randomly selected to determine their antimicrobial susceptibility phenotype, indicating that those isolates were multidrug-resistant and displayed a typical ESBL phenotype. In addition, the non-ESBL *bla*_TEM-1b_ and a novel chromosomal variant of *bla*_SHV_ were detected by PCR and amplicon sequencing and confirmed by WGS. Previous studies also reported the presence of non-ESBLs *bla*_TEM_ and *bla*_SHV_ allelic variants in *K. pneumoniae* strains in clinical and non-clinical isolates including water, soil and animals raised for food production (Liakopoulos et al., 2016b; Shahraki-Zahedani et al., 2016). In addition to beta-lactams, it is concerning that resistance genes to other antibiotic classes including fluoroquinolones, tetracycline and aminoglycosides were also detected here, but considering results of a recent study in human *K. pneumoniae* isolates in the Netherlands, our results are not surprising (Hendrickx et al., 2020).

Several studies reported that the dissemination of the *bla*_CTX-M-15_ gene among Enterobacterales has been facilitated by the IncFII plasmids (Peirano and Pitout, 2010; Mansour et al., 2015; Stercz et al., 2021). In our study we showed that the *bla*_CTX-M-14_ gene dissemination was facilitated by the IncFII_K_ and IncFIB_KN_ plasmid replicons in four ESBL-*K. pneumoniae* isolates tested. Previous studies showed the presence of *K. pneumoniae* isolates harboring *bla*_CTX-M-14_ located on IncFIB_K_ and IncFII_K_ obtained from healthy red kangaroos (Wang et al., 2020) and from a clinical isolate in China (Zhang et al., 2017). Likewise, the spread of the *bla*_CTX-M-14_ gene in Enterobacterales has been predominantly associated with IncFII and IncK plasmid replicons in humans and animals including cattle in several countries (Hou et al., 2012; Stokes et al., 2012).

Several clonal lineages of *K. pneumoniae* have been reported widely in the United States and Europe such as ST258 and are currently present in several European countries (Woodford et al., 2011; Rodrigues et al., 2014; Ríos et al., 2017), supporting the epidemic potential of these clones. Our results showed that ST107 was the only ST type identified among ESBL-*K. pneumoniae* isolates in a single veal farm. The *K. pneumoniae* ST107 has been previously identified in *K. pneumoniae* isolates harboring *bla*_KPC-2_ from a hospital environment (Fu et al., 2018), *K. pneumoniae* producing NDM (NDM-9) from a human clinical isolate in China (Wang et al., 2014), and in *K. pneumoniae* isolates harboring *bla*_SHV-11_ from pigs and *bla*_CTX-M-1_ from cattle (Klaper et al., 2021).

We aimed to determine if the isolates were clonally related by calculating the pairwise Single-Nucleotide Polymorphism (SNPs) distances based on the core genome predicted genes. The high number of SNPs detected exclude the possibility to confirm this hypothesis (data not shown). However, it seems that these isolates may have been present at the farm beyond the time of a single production round. To confirm this hypothesis additional sampling of the farm, a bigger sample size and molecular analysis will need to be performed.

The low diversity of ESBL-PE reported in this study and the clonal spread of ESBL-*K. pneumoniae* during the rearing period highlight the importance to reinforce the hygiene and management practices in farms as previously reported (Bokma et al., 2019; Damiaans et al., 2019). Recently, Atterby et al. (2019) showed that direct contact with animal manure and animal slaughter products are potential risk factors for fecal carriage of ESBL-*E. coli* and ESBL-*K. pneumoniae* in humans and animals. Additionally, the authors indicated that daily removal of animal manure could decrease the environmental exposure to antibiotic-resistant bacteria (Atterby et al., 2019). In the current study, the fecal carriage of ESBL-*K. pneumoniae* started to spread rapidly from week 6 after the calves were transferred from the dairy farm to the veal farm, suggesting that the ESBL-*K. pneumoniae* originated from the veal farm environment, although colonization of one of the animals before transport from the dairy to the veal farm or contact with other calves or animals present in the farm cannot be ruled out. While the pathogen that was responsible for clinical respiratory problems on this farm has not been investigated, *K. pneumoniae* could have caused these symptoms while the usage of antimicrobials may have increased transmission. As such, an increase in hygiene measures on farms in which respiratory disease is seen could lead to a decrease in intestinal colonization which in turn could lead to a decrease in environmental contamination. We also hypothesis that the administration of nonsteroidal anti-inflammatory drug (NSAID) for at least 2 weeks continuously in combination with antibiotics to control the respiratory infection, could perhaps contribute to the presence of *K. pneumoniae* in feces because the use of this drug can cause gastrointestinal upset such as irritation, therefore affecting the colonization resistance population present in the gastrointestinal tract. Further research is needed to better understand the relationship between animals, farmworkers, and the environment to further assess and limit the exposure and risk of fecal carriage of antibiotic-resistant bacteria.

## Data Availability Statement

The datasets presented in this study can be found in online repositories. The names of the repository/repositories and accession number(s) can be found at: https://www.ebi.ac.uk/ena, PRJEB50519.

## Ethics Statement

The animal study was reviewed and approved by The Central Committee on Animal Experiments (the Hague, Netherlands; approval number 2017.D-0029). Written informed consent was obtained from the owners for the participation of their animals in this study.

## Author Contributions

MB and KvR designed the study. FM collected the samples and data. TB performed the sample analysis, processed the data, and wrote the manuscript. AK and QD contributed with the laboratory work. MB, KV, FM, and KvR contributed to review and editing the manuscript. All authors contributed to the article and approved the submitted version.

## Funding

The collection of isolates that were used for this study was funded by the Dutch Ministry of Agriculture, Nature and Food Quality grant BO-43-111-011. Funding for the analysis of the isolates was received from the European Union’s Horizon 2020 research and innovation programme through One Health EJP Project Full-Force [grant agreement number 773830] with co-funding from TKI bureau AgriFood.

## Conflict of Interest

The authors declare that the research was conducted in the absence of any commercial or financial relationships that could be construed as a potential conflict of interest.

## Publisher’s Note

All claims expressed in this article are solely those of the authors and do not necessarily represent those of their affiliated organizations, or those of the publisher, the editors and the reviewers. Any product that may be evaluated in this article, or claim that may be made by its manufacturer, is not guaranteed or endorsed by the publisher.

## Supplementary Material

The Supplementary Material for this article can be found online at: https://www.frontiersin.org/articles/10.3389/fmicb.2022.866674/full#supplementary-material

## Footnotes

^1^ https://www.eurl-ar.eu/protocols

^2^ www.eucast.org

^3^ www.genomicepidemiology.org

^4^ www.pubmlst.org
